# Alcohol in Mouth Disinfection

**Published:** 1907-08-15

**Authors:** W. F. Eitch


### ALCOHOL IN MOUTH DISINFECTION.

BY W. F. EITCH, D. D. S.

The mouth as the chief entrance way to the respiratory and digestive tracts is necessarily exposed to constant invasion by all air-borne organisms as well as by those conveyed in food and drink. Such organisms find ready lodgment in the mucous folds, dental sulci, interspaces and other harboring crevices; while in the oral secretions and body temperature are conditions favoring growth and development.

Many of these organisms have no special pathological significance; others on the contrary, are specifically infective, and while in the mouths of those whose resisting power to disease is adequate they may remain dormant for a time, to finally degenerate or be destoyed by phagocytic action; others, under conditions more favorable for their propagation, become infective foci of disease in the oral cavity the pulmonary or digestive tracts, or though general invasion of the circulatory system.

While even among primitive races the removal from the mouth of foreign substances in their grosser forms has usually been regarded as an obligatory feature of the daily toilet, any serious effort at systematic daily disinfection of the mouth, through the removal or destruction of micro-organisms, has necessarily been reserved for the present period, as not until now has their existence and causative relation to diseae been recognized and demonstrated. Oral hygiene or "prophylaxis" as practiced to-day is in a large measure directed to their inhibition or elimination. Hence an infinite number of powders, pastes and washes more or less antiseptic in character are now in use as adjuncts to the scouring qualities of the tooth brush.

Under these circumstances the question of the real efficacy of such preparations, and of the efficacy of any known method or system ot oral disinfection, is ot no little importance, both in the interests of exact science and of the public health. Prof. Miller, who was the first to make exact and extended experimental research regarding the possibilities of mouth disinfection by the use of antiseptics, and Rose who more recently (1901) published the results of his own studies in the same field, have found complete mouth disinfection impossible with any antiseptic or disinfectant the continued employment of which in sufficient strength would be permissible.

A still more recent investigation is that of Augustus Wadsworth, M. D., instructor in bacteriology and hygiene in the College of Physicians and Surgeons, New York, the results of whose extended experiments are embodied in a paper, entitled "Mouth Disinfection in the Treatment and Prophylaxis of Pneumonia" published in the *Journal of Infectious Diseases*, October, 1906.

By the nature of his research Dr. Wadsworth's investigations were necessarily confined to one special organism, the pneumococcus. Concerning this organism he finds that beyond all other pathogenic bacteria, with the possible exception of the streptococci, it is more apt to become established in the mouth as a harmless parasite, which, however, does not usually long survive in the purely mucous secretion of the healthy, intact mucous membrane; but he finds that, being extremely susceptible to environmental change, the pneumococcus often rapidly acquires an "exalted virulence."

A significant result of Dr. Wadsworth's experiments with the pneumococcus was that disinfecting agents which proved effective in laboratory tests with broth cultures were, owing to the protective action of the mucous secretions, quite ineffective when applied to the same organisms in the mouth.

Briefly stated, the results obtained by Dr. Wadsworth with various antiseptics were as follows: The germicidal powers of mercuric chlorid and phenol he found to be much impaired by their chemical combination with and precipitation of albuminous substances, the bacteria being enmeshed and protected by the coagula. Hydrogen peroxid and formalin, while quickly destroying organisms in broth cultures, failed to act in mouth exudates. All of the most widely known proprietary antiseptic preparations, of which listerine is the type, were tried with negative results. Potassium permanganate does not appear to have been tested; probably the result would have been the same as with other oxidizing agents.

A simple saline solution, containing 0.5 per cent. of sodium chlorid and 0.25 per cent. of sodium bicarbonate, was experimented with as a mouth wash for patients in hospitals with pneumonia, and the effect tested by injecting their sputum, collected after the washing, into mice, which in every instance died from pneumococcus infection. Similar tests were made with alcohol, recommended by Rose as the most efficient antiseptic in the mouth when used with the tooth brush. Rose employed 60 per cent. alcohol, but, owing to its hardening and desiccating effect upon the tissues when of that strength, 30 per cent. was the strength adopted by Dr. Wadsworth in his experiments.

The results with alcohol in pneumonia cases were the same as those secured after the use of saline washes: mice injected with the sputum all died; the only difference being that the deaths occured "at more irregular intervals." These results demonstrated that in every case the mouth disinfection was incomplete and that "rinsing and gargling the mouth and pharynx, as practiced in hospital routine, is a useless procedure so far as getting rid of the virulent pneumococci is concerned."

For the destruction of the non-virulent pneumococci in the mouths of healthy persons the mild saline solution was found quite as ineffective as it had proved for the virulent type. With alcohol, however, the result in healthy mouths was more hopeful. After its thorough use pneumococci could not be obtained from the mouth secretions, and Dr. Wadsworth concludes that "removing and disinfecting the contaminated secretions from the more accessible parts of the mouth may in time accomplish the elimination of pneumococci from healthy persons."

This conclusion, in some measure, justifies the growing confidence in alcohol as a mild, safe and efficient antiseptic agent. In ancient medicine it was held in high repute as a vulnerary, and has been too much neglected in the general surgical as well as the dental practice of our own time.

While Dr. Wadsworth regards alcohol as the most effective of all agents for general mouth disinfection, he states that his best results were obtained by combining it with salines and glycerin and with enough thymol, menthol and aromatic oils to flavor the mixture. The following is his formula:

℞ Sodium chlorid (c. p.)---------------ʒss.<br>

Sodium bicarbonate (c. p.)----------gr. x<br>

Water (dist)-------------------------ʒij<br>

Alcohol -----------------------------℥v<br>

Glycerin-----------------------------℥j<br>

Thymol }<br>

Menthol } ----------------------aa.gr.ij<br>

Oil of Wintergreen ---------------gtt.iij<br>

Oil of cinnamon ------------------gtt.ij<br>

Oil of eucalyptus-----------------gtt.v<br>

Tr. cudbear----------------------ʒjss<br>

Tr. rhatany-----------------------ʒss<br>

M. Sig. Dilute with water equal parts.

In preparing this solution the salts should first be dissolved in the water before adding the alcohol.

In the experience of the present writer saline solutions in the mouth and associated cavities are chiefly valuable for their effect upon the mucous secretions, the viscidity and adhesiveness of which they greatly lessen, thus favoring their detachment from the mucous membrane and leaving its surface open to the action of whatever antiseptic may be employed. In antral empyema, especially, a saline douche is of great service as a preliminary to injection with any antiseptic with which the salines are not chemically incompatible. For mouth disinfection thorough lavation with saline solutions, followed by a strong alcoholic wash, would probably be an even more effective procedure than the combination of salts and alcohol called for by Dr. Wadsworth's formula.—*Dental Brief.*

